# Finding on a chest radiograph: A dangerous complication of subclavian vein cannulation

**DOI:** 10.4103/0972-5229.68225

**Published:** 2010

**Authors:** Nataraj Madagondapalli Srinivasan, Akshay Kumar

**Affiliations:** **From:** Department of Anaesthesiology, Kasturba Medical College, Manipal - 576 104, India

**Keywords:** Central venous catheters, complication of subclavian vein cannulation

## Abstract

Cannulation of the subclavian vein has its inherent risks. Post procedure chest radiograph is one of the investigations done to rule out immediate complications. Unless the clinician is aware as to what to look for in the radiograph, some of the dangerous complications can be overlooked. Accidental subclavian artery cannulation is identifi ed immediately by color and jet of the blood. Also the position of the catheter tip has to be confi rmed by obtaining the arterial pressure tracing using a pressure transducer. Non availability of Doppler ultrasound and pressure transducer are limiting factors for immediate confi rmation of proper catheter placement. Also, in patients with severe hypotension and reduced oxygen content of blood, accidental arterial puncture may not show the characteristic bright red pulsatile back fl ow of arterial blood. In these situations radiography can be used as a diagnostic tool to rule out subclavian artery cannulation.

## Introduction

Subclavian vein cannulation is a commonly performed procedure in critical care units for several reasons like good venous access, hemodynamic monitoring, parenteral nutrition, etc.

Even though it is a relatively simple procedure in well trained hands, it is not without complications which can sometimes be devastating. Many times the complications are immediately recognized and steps are taken to rectify them. When central venous catheters are inserted without ultrasound guidance or when pressure transducers are not available immediately to confirm the pressure tracings, it requires a great degree of suspicion and ability to pick up subtle findings on a radiograph to minimize the consequences of unidentified complications.[1] We present a case of accidental subclavian artery cannulation when attempting right subclavain vein cannulation which could be picked up on a chest radiograph as the catheter tip crossed the midline and was located on the left side of the chest. This subtle finding is important because in patients who are severely hypovolemic and have poor oxygenation, differentiating arterial from venous blood backflow is made difficult either due to low systemic pressure and/or reduced oxygen content of the blood. Also in emergency situations where accessibility to a Doppler probe and pressure transducer is difficult, ruling out accidental arterial cannulation can be difficult.

## Case Report

A 28 year female patient was admitted with fever of five days duration and difficulty to breathe since one day. On physical examination, she was in respiratory distress, requiring oxygen supplementation. Her heart rate was 120 beats/ min, radial pulse was feeble, blood pressure 80/40 mmHg, respiratory rate 30/min, and SpO_2_ 85%.

**Figure 1:**
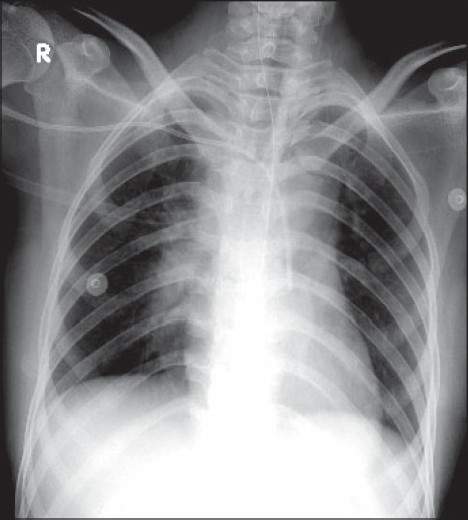
Radiograph of chest showing the tip of the catheter on the left side of the chest

Multiple attempts at peripheral vein cannulation had failed. A right sided subclavian vein catheter was planned in view of her general condition and need for fluid and inotropic support. A 15 cm 7 Fr triple lumen central venous catheter was inserted into the right subclavian vein using the Seldinger technique on first attempt with no difficulty. A good back flow of blood was also confirmed. Quick look at the post procedure chest radiograph confirmed the position of the catheter and absence of pneumothorax [Figure 1]. Initial central venous pressure (CVP) tracing could not be obtained due to non availability of a transducer. On connecting the intravenous (IV) fluid infusion to one of the lumens of CVP catheter, there was no free flow of the IV fluid.

Possibility of a subclavian artery cannulation was suspected based on a re-examination of the chest radiograph which showed the catheter crossing the midline and tip located on the left side [Figure 1].

Hence the catheter was removed immediately. There were no complications but for prolonged bleeding from the puncture site which required pressure for about fifteen minutes.

## Discussion

Use of subclavian veins for CVP monitoring, fluid and blood products administration, and hyperalimentation has become very popular. Even though the infraclavicular approach for subclavian vein catheterization is a relatively simple technique, it is not without complications.[2]

Accidental puncture or cannulation of the subclavian artery are not common but are easily recognised clinically by a rapid pulsatile bright red back flow of blood and also difficulty in administering IV fluids.[3] Use of Doppler ultrasound for cannulation reduces the complications like arterial puncture. Also, obtaining the arterial pressure tracing using a pressure transducer confirms the arterial placement. During immediate non availability of these facilities, the clinician performing the subclavian vein cannulation has to depend on the radiograph finding which can be obtained easily and is relatively cost effective.

Knowledge of the anatomy of the subclavian area is essential for a successful cannulation of subclavian vein. The vein is found within the costoclavicular-scalene triangle. The axillary vein becomes the subclavian vein at the lateral border of the first rib. It then traverses over the first rib and behind the medial third of the clavicle while resting on the apical pleura, joins the internal jugular vein to form the innominate and subsequently forms the superior vena cava. It normally remains on the right side of the mediastinum.

The right subclavian vein does not cross the midline, but the left subclavian vein does.[3] In our case, even though the CVP catheter crossed the midline and was obviously in the artery (as it can be seen going down to aorta in Figure 1) it was not recognised by the untrained eye. The central point of this case report is that, one should not only look for the pneumothorax on a chest radiograph, but also the position of the catheter as well.

The structures most often injured by improper insertion of the needle include the posterior wall of the vein, the apical pleura, the underlying lung, the subclavian artery, the brachial plexus, the phrenic nerve, etc. The complication rate varies from 0 to 9.9% and is higher when inexperienced individuals perform the catheterization.[4] The subclavian artery lies above and posterior to the vein. Because the artery originates from the brachiocephalic artery and aorta, a catheter inserted in the right subclavian artery will be above the clavicle and traverses across the midline.[5]

## Conclusion

If the right subclavian central venous catheter is seen crossing the midline on a chest radiograph, arterial cannulation should be suspected and steps taken to determine arterial placement.

## References

[1] Wilson JN, Grow JB, Demong CV, Prevedel AE, Owens JC (1962). Central venous pressure in optimal blood volume maintenance. Arch Surg.

[2] Borja AR (1972). Current status of infraclavicular subclavian vein catheterization: review of English literature. Ann Thorac Surg.

[3] Dedhia HV, Schiebel F (1987). What is wrong with this chest roentgenogram?. Right subclavian artery cannulation. Chest.

[4] Mansfield PF, Hohn DC, Fornage BD, Gregurich MA, Ota DM (1994). Complications and failures of subclavian-vein cannulation. N Engl J Med.

[5] Civetta JM, Shoemaker WC, Thompson WL (1980). Fullerton CA: Society of Critical Care Medicine. Critical care state of the art, vol 1, chapter 13. Fullerton CA: Society of Critical Care Medicine.

